# Supplementary material for: Are carbon emissions trading and green financial instruments synergistic? -Comprehensive quantitative research based on content analysis
Source: PLoS One. 2024 Mar 7;19(3):e0298601. doi: 10.1371/journal.pone.0298601 (PMC10919601; doi:10.1371/journal.pone.0298601)
Supplement: S1 Data — (ZIP) [file pone.0298601.s001.zip › Data/地级市Z_F工作报告.zip!/╣≤╓▌/╣≤╤⌠╩╨/└┤╫╘╣1⁄2╓┌║┼í╛┬φ┐╦╩2╛▌═°í┐_2016.docx]

各位代表：

　　现在，我代表市人民政府向大会作工作报告，请予审议，并请市政协委员和其他列席人员提出意见。

　　一、“十二五”时期工作回顾

　　“十二五”以来，在省委、省政府和市委的坚强领导下，在市人大、市政协的监督支持下，我们牢牢守住发展和生态两条底线，紧扣主基调、实施主战略，大力推进“六大工程”，全面打造贵阳发展升级版，顺利完成“十二五”规划目标任务，率先在全省建成全面小康社会，经济社会发展实现历史性新突破。

　　“十二五”是我市奋力后发赶超、增比进位，综合实力大提升的五年。与“十一五”末相比，预计全市生产总值从1121.82亿元提升到2890.71亿元，年均增长15.1%（按可比价计算）。全社会固定资产投资从1019.1亿元提升到4015亿元，年均增长31.6%。社会消费品零售总额从484.78亿元提升到1060.4亿元，年均增长15.4%。外贸进出口总额从26.42亿美元提升到111亿美元，扣除服务贸易年均增长28.1%。财政总收入从304.64亿元提升到723.3亿元，年均增长18.9%。公共财政预算收入从136.3亿元提升到374.15亿元，年均增长22.4%；税收收入占公共财政预算收入比重保持在80%以上，收入质量在全省保持最优。公共财政预算支出从204.38亿元提升到503.59亿元，年均增长19.8%。城镇、农村居民人均可支配收入分别从16597元、5976元提升到27266元、11972元，城乡居民收入比从2.78：1缩小到2.28：1。万元生产总值能耗累计下降18%，节能减排完成省下达的目标任务。五年来，经济增速在全国省会城市中4年位居第一、2012年位居第二，经济总量占全省比重从24.4%提高到27.5%，首位度提高3.1个百分点，在全省增比进位综合测评中除2012年外4年位居第一。

　　“十二五”是我市致力创新驱动、转型升级，产业结构调整大突破的五年。京筑创新驱动区域合作成果丰硕，中关村贵阳科技园精彩亮相、引领转型，抢占大数据发展先机，寻找大数据产业“蓝海”，城市对外影响力大幅提升。以高新技术和现代制造业为龙头的现代工业、以金融业为龙头的现代服务业、以都市农业为龙头的现代农业蓬勃发展，三次产业结构比例从5.1：40.7：54.2调整为4.1：38.3：57.6。大数据产业从无到有，在全国实现“九个率先”，数据中心服务器达2.5万台，呼叫中心坐席突破10万席，大数据及其关联产业规模总量近千亿元。规模以上工业增加值突破700亿元，高新技术产业占比达40%。贵州金融城基本建成，贵阳互联网金融特区挂牌投用，金融机构存贷款余额分别达8772.2亿元和7875.6亿元，金融业占经济总量比重达10.8%，贵·电商平台建成投用，电子商务交易额突破500亿元，会展经济进入全国省会城市前列，物流业增加值达170亿元，旅游总收入年均增长26.5%。现代农业增加值占一产增加值比重提高到30%，成功举办首届农业嘉年华活动。“5个100工程”完成投资3906.82亿元，小孟工业园、沙文生态科技产业园等一批重点产业园区相继建成，集聚功能不断增强。

　　“十二五”是我市竭力坚守底线、示范引领，生态文明大发展的五年。获批建设全国首个生态文明示范城市并取得阶段性成效，“蓝天”“碧水”“绿地”“清洁”“田园”五项保护计划有力推进，环境空气质量优良率90%以上，集中式水源地水质达标率100%，森林覆盖率达45.5%，建成区人均公共绿地达10.95平方米，新建花溪十里河滩、阿哈湖等国家级城市湿地公园，完成南明河水环境综合整治一期工程，获批全国水生态文明建设试点城市，全国首批节能减排财政政策综合示范工作顺利通过验收。生态文明体制机制走在全国前列，首创成立生态文明建设委员会，组建生态保护“两庭三局”，率先出台《贵阳市建设生态文明城市条例》，实行生态环境损害党政领导干部问责等系列政策措施。“爽爽的贵阳”享誉国内外，成为我们的“金字招牌”。

　　“十二五”是我市大力突破瓶颈、优化功能，城乡面貌大变化的五年。城乡规划体系形成“规划树”层级结构，“双核多组团”城市空间布局逐步形成，城乡基础设施建设实现重大突破，“疏老城、建新城”成效明显。“三环十六射”骨干路网全面建成，1.5环、BRT（快速公交）、轨道交通1号线和2号线、西南商贸服务业集聚区综合交通枢纽启动建设、加快推进，龙洞堡国际机场跻身国内千万级大型繁忙机场行列，高速公路通车里程近500公里、对外通道达8个，贵广高铁和市域快铁改貌货运场、久永货运专线等开通运营，贵阳进入高铁时代并融入全国7小时经济圈。五里冲批发市场、花溪大道汽车市场、市西路批发业态等实现整体转移，孔学堂、筑城广场、城乡规划展览馆等功能性设施建成投用，累计完成棚户区城中村改造项目183个，城市建成区面积达360平方公里，较“十一五”末增加约130平方公里。建成“美丽乡村”提高型示范点120个、示范带10条，开工建设渔洞峡等中小型水库16座,行政村通村沥青（水泥）路通畅率、客运通达率均达100%。荣获全国文明城市、国家卫生城市称号，创建国家环境保护模范城市顺利通过验收，成功协办第九届全国少数民族传统体育运动会，城市对外形象大幅提升。

　　“十二五”是我市努力深化改革、扩大开放，活力动力大增强的五年。重点领域和关键环节改革取得新突破，非公有制经济占生产总值比重从47%提高到51%，各类企业注册资本突破万亿元，市级行政审批事项从93项减少到67项，“数据铁笼”试点成功，大数据综合治税系统启动建设，“六统一”土地出让和供给制度全面施行，市属平台公司向市场化转型发展，农村产权制度改革走在全省前列，新一轮行政区划调整和新一轮政府机构改革全面完成，建立“借、用、还”一体化投融资运行机制，有效防控政府性债务风险，实现“有钱运转、有钱还债、有钱发展”。对外开放平台和载体建设迈上新台阶，形成“四轮驱动”开放新格局，贵阳综保区一年通过国家验收、当年实现封关运行，航空港经济区一年拉出总体骨架，高新区、经开区作为中关村贵阳科技园核心区一年亮出新形象，生态文明贵阳会议升格为生态文明贵阳国际论坛，高标准举办数博会、众筹大会、电商峰会、中国——东盟教育周、酒博会等重大活动，与美国、爱尔兰、加拿大、韩国等国家有关城市建立友好城市关系，五年累计引进省外到位资金8000亿元，实际直接利用外资30.72亿美元，年均分别增长44.7%和47.1%。

　　“十二五”是我市全力改善民生、增进福祉，人民生活水平大提高的五年。持续办好民生实事、有效化解“民生十困”，财政对民生的投入累计达1207.31亿元，年均增长22.2%。38.3万贫困人口、432个贫困村“脱贫摘帽”，在全省率先消除绝对贫困。累计新增城镇就业89.21万人，城镇登记失业率控制在4.5%以内。实施“教育立市”战略，农村义务教育阶段学校营养餐改善计划实现全覆盖，新建改建幼儿园和中小学校677所，新增学位8.7万个，与北京等地优质学校合作办学34所，贵州（清镇）职教城入驻院校19所，“一本三专”高等教育格局不断提升，师德、师风建设进一步加强，涌现出刘芳等先进教育工作者。城乡居民大病保险实现全覆盖，新建改建乡镇卫生院60所，完成中心乡镇卫生院标准化建设，启动城市公立医院综合改革，成立市医院管理（集团）有限公司，县级公立医院综合改革全面实施。城乡低保、基本医疗、基本养老等保障标准不断提高，农村留守儿童、困境儿童和留守老人关爱救助保护实现全覆盖。实施公租房建设10.69万套，改造农村危房2.3万户，完成“三年千院”行动计划项目2431个。向“用水难”宣战，城区饮水困难的127个小区28万余人用上质量洁净、供应稳定的自来水，新建农村饮水安全工程516处，新增解决农村饮水安全35.91万人。加大城市农副产品“保供稳价”力度，建成1万亩高标准蔬菜保供基地和4个二级批发市场、一批生鲜直销超市。加快“新型社区·温馨家园”建设，启动“社会和云”工程，推进网格化精细化管理，城乡社会治理体系和治理能力不断提升。狠抓社会治安、信访维稳、安全生产，刑事发案数、万人发案数分别比2010年下降38.4%和49.6%，信访案件化解率达97%，安全生产事故起数、死亡人数分别下降53%和44.4%，各类应急突发事件得到妥善处置，群众安全感达97.38%。

　　过去的五年，文化体育和其他社会事业长足进步，“三足鼎筑”精神大厦初现雏形，创建花溪文化旅游创新区成效明显，成功举办2015贵阳国际田联世界越野锦标赛、贵州省第九届运动会等重大赛事。国防、人防、国家安全、食品药品安全、审计、司法行政、民族宗教、移民、地方志、档案、侨务、对台、仲裁、气象、老龄、工青妇、关心下一代、慈善、残疾人、红十字、爱国卫生等事业全面发展、成效显著。

　　过去的五年，我们不断加强政府自身建设，着力打造法治政府，提请市人大常委会审议地方性法规草案13件，制定政府规章70件，办理人大代表建议935件、政协提案1892件，办复率均达100%。扎实开展党的群众路线教育实践活动和“三严三实”专题教育，深入找问题、找差距、找目标，着力抓统筹、抓落实、抓提升，不断提高政府执行力、服务力、公信力。建立权力清单和责任清单制度，严控“三公”经费，严厉查处各类违规违纪违法行为，勤政廉政建设取得新成效。

　　刚刚过去的2015年，我们积极适应经济发展新常态，保持定力，沉着应对，综合施策，精准发力，完成年初确定的主要目标任务，经济社会发展稳中有进、稳中有新、稳中向好。

　　一年来，我们倍受关怀、深受鼓舞，坚定了后发赶超的信心和决心。习近平总书记亲临贵阳视察指导，充分肯定我们的工作，要求我们守底线、走新路，为我们提供了理论指导和行动指南。李克强总理等党和国家领导人先后视察贵阳，为我们带来特别的关爱和支持，马凯、汪洋、孟建柱、郭声琨等领导同志对我市大数据、社会治理、禁毒工作等作出指示批示，提出表扬。省委、省政府和赵克志书记、陈敏尔书记、孙志刚代省长对贵阳高看一眼、厚爱一层，多次表示对贵阳的工作是满意的、放心的，充分肯定我们有新的思路、新的路径、新的增长、新的高度、新的面貌。省委常委会专题听取贵阳工作情况汇报，明确提出贵阳要打造创新型中心城市，为我们未来发展精准把脉、指明方向。中央和国家部委、省直部门对贵阳工作高度认同，在发展大数据产业、生态文明建设等方面积极帮助出谋划策，给予大力支持。

　　一年来，我们苦干实干、奋力冲刺，确保了“十二五”胜利收官。预计全年实现生产总值2890.71亿元、增长12.5%，在全国省会城市中经济增速继续位居第一，在全省增比进位综合测评中继续排名第一，白云区、云岩区、南明区、息烽县、观山湖区位居全省31个经济强县（市、区）前十。从三次产业看，一二三产协调发展，一产增加值增长6.1%，二产增加值增长14.5%，三产增加值增长11.1%。从三大需求看，投资、消费、出口持续发力，完成500万元口径固定资产投资2757亿元、增长18%，社会消费品零售总额1060.4亿元、增长11.5%，外贸进出口总额111亿美元、增长41.5%。从三个收入看，财政、企业、城乡居民稳步增收，公共财政预算收入374.15亿元、增长12.8%，剔除结构性减税及政策性减免税费因素后增长14.1%，公共财政预算支出503.59亿元、增长12.25%；规模以上工业企业利润总额225亿元、增长16%；城乡居民收入分别达27266元和11972元、增长9.2%和10.6%。在《经济学人》智库发布的《2015年中国新兴城市报告》中，贵阳从5年前的第65名上升为第1名。

　　一年来，我们弯道取直、创新突破，办成了一批标志性的大事。成功举办全球首个以大数据为主题的博览会和峰会、生态文明贵阳国际论坛2015年年会、首届众筹大会。在全国率先组建大数据交易所等引领性平台，获批创建国家级大数据产业发展集聚区和大数据产业技术创新试验区，英特尔众创空间加速器、戴尔——高新翼云IT联合实验室、博科——高新翼云网络交换技术实验室挂牌成立，谷歌、百度等国际知名企业相继入驻，吉利新能源汽车等重大项目落地建设，贵阳银行IPO上市获中国证监会无条件通过。四大开放平台建设取得突破，航空港经济区建设起步之年成效显著，正威大宗商品交易中心建成运营。贵广高铁实现“公交化”运行，沪昆高铁贵州东段、市域快铁贵开线和白云至龙里联络线开通营运。社会治安立体防控体系进一步形成，禁毒工作经验做法得到中央政法委、公安部充分肯定，群众安全感排位由全省第3名上升到第2名。环境空气质量优良率达93.2%，在全国74个重点城市排第8位。

　　一年来，我们知行合一、协力争先，提振了干事创业的精气神。以陈刚书记为班长的市委坚强领导、正确决策，统揽全局、协调各方，保持战略定力，增强发展韧劲，团结带领全市干部群众拼搏奋斗、攻坚克难，一心一意谋发展、聚精会神搞建设，全市上下干事创业的激情空前高涨，筑城大地呈现“发展要快、风气要正、作风要实、干部要干”的良好政治生态。所有这些，汇聚了加快发展的正能量，发出了“爽爽的贵阳”的好声音！

　　各位代表！过去五年是贵阳经济社会发展最好最快的时期之一，五年来取得的巨大成就，充分证明市委各项决策部署是完全正确的，只要我们把思想统一到发展上来、把心思集中到发展上来、把力量凝聚到发展上来，完全可以走出一条西部欠发达城市经济发展与生态改善“双赢”的可持续发展之路。五年来的发展启示我们，必须始终坚持吃透上情、掌握市情、把握外情，在解放思想中走符合贵阳实际的发展路径；必须始终坚守两条底线，既要“绿水青山”，又要“金山银山”，推动两条底线一起守、两个成果一起收；必须始终坚持改革开放创新，以改革促进开放、以开放倒逼改革，为经济社会发展提供持久动力；必须始终坚持一切为了人民的价值取向，切实实现好、维护好、发展好最广大人民群众的根本利益；必须始终狠抓落实，坚决做到党委有号召、政府有行动，党委有要求、政府有落实，努力做好市委正确决策的“证明题”。

　　各位代表！以上成绩和经验的取得，是省委、省政府和市委正确领导的结果，是省直部门和兄弟市（州）大力支持的结果，是市人大、市政协监督支持、共同努力的结果，是全市干部群众团结奋斗、苦干实干的结果。在此，我代表市人民政府，向全市各族人民，向各位人大代表和政协委员，向各民主党派、工商联、无党派人士、各人民团体和各界人士，向驻筑人民解放军、武警官兵和人民警察，向中央及省在筑单位，向市法院、市检察院，以及所有关心、支持贵阳建设发展的各位老领导、老同志和各界朋友，表示衷心的感谢!

　　成绩令人鼓舞，问题不容忽视。我们也清醒地看到工作中存在的困难和不足：一是宏观经济下行压力较大，个别经济指标增速放缓，部分企业生产经营困难；二是产业发展“青黄不接”，标志性的大产业、大企业、大项目缺乏，大数据先发优势和发展基础还不牢固；三是城乡区域发展不协调，城市“脏乱差”未得到根本解决，农村水电路房气讯等基础设施薄弱，脱贫标准不高；四是城市承载能力不足，配套、运行和管理体系不够完善，资源环境约束趋紧；五是民生保障能力有待提高，优质教育、医疗资源不足，交通拥堵等问题仍未有效缓解；六是干部能力和作风与“作表率、走前列、做贡献”、与当好“火车头”“发动机”的要求仍有差距。针对这些问题，我们将在今后工作中采取有效措施，切实加以解决。

　　二、“十三五”时期的主要目标任务

　　“十三五”时期是我市守底线、走新路、打造升级版的关键时期。我们既要看到欠发达欠开发的基本市情没有变，既要“赶”又要“转”的双重任务没有变，处于西部省会城市第三梯队的状况没有变，更要看到，我们拥有中央和省委、省政府给予的利好政策支撑、大数据发展先行先试的实践支撑、西南交通枢纽的区位支撑、众多国际性活动的平台支撑、清爽空气与凉爽天气的生态支撑、干部群众不甘落后的动力支撑。站在新的历史起点上，我们要牢牢把握认识新常态、适应新常态、引领新常态这个大逻辑，以打破才能得生机的勇气、取直自然出新路的智慧、实干一定有收获的定力，思想一致、步调协调，勇担使命、倾力奉献，加快实现经济社会发展历史性新跨越。

　　“十三五”时期我市经济社会发展的总体要求是：高举中国特色社会主义伟大旗帜，以马克思列宁主义、毛泽东思想、邓小平理论、“三个代表”重要思想、科学发展观为指导，深入贯彻习近平总书记系列重要讲话精神，全面贯彻党的十八大和十八届三中、四中、五中全会，省委十一届六次全会和市委九届五次全会精神，按照“四个全面”的战略布局，贯彻创新、协调、绿色、开放、共享五大发展理念，牢记“作表率、走前列、做贡献”的历史使命，坚持主基调、主战略，率先落实大扶贫、大数据两大战略行动，突出抓好“产业发展要有新业态、城乡建设要有新形态”两项重点任务，以“守底线、走新路、打造升级版”为总揽，培植后发优势、实现弯道取直、奋力后发赶超，全力打造创新型中心城市，建成大数据综合创新试验区、全国生态文明示范城市、更高水平的全面小康社会，在全省发展中当好高铁路上的“火车头”和战斗机上的“发动机”。

　　“十三五”时期我市经济社会发展的战略路径是：坚持改革创新、精准发力，纵深推进“六大工程”，打造贵阳发展升级版。一是纵深推进科技引领工程，打造创新贵阳升级版；二是纵深推进平台创优工程，打造开放贵阳升级版；三是纵深推进公园城市工程，打造生态贵阳升级版；四是纵深推进文化惠民工程，打造人文贵阳升级版；五是纵深推进社会治理工程，打造法治贵阳升级版；六是纵深推进凝心聚力工程，打造和合贵阳升级版。

　　“十三五”时期我市经济社会发展的主要预期目标是：生产总值年均增长11%左右，总量突破5000亿元，占全省的比重持续提高；工业规模总量实现翻番；固定资产投资年均增长16%；社会消费品零售总额年均增长11%；外贸进出口总额年均增长11%；公共财政预算收入年均增长11%；城镇、农村常住居民人均可支配收入分别突破43000元和20000元，农村低收入困难群体人均可支配收入整体越过6500元；森林覆盖率达到50%以上，集中式饮用水源地水质达标率稳定在100%，空气质量稳定在全国省会城市前列。

　　打造创新型中心城市，建成大数据综合创新试验区、全国生态文明示范城市、更高水平的全面小康社会，是市委九届五次全会确定的“十三五”奋斗目标，是我们践行五大发展理念的实际举措，既符合国家战略又体现地方特色和时代特征，既符合战略目标又体现战略部署和战术执行。打造创新型中心城市是战略核心，对“三个建成”具有统揽和引领作用，以大数据、大生态、大扶贫为关键词的“三个建成”，是三个战略支撑，构成了贵阳打造创新型中心城市的核心内容。

　　围绕“一个目标、三个建成”，我们将突出“六个更加注重”，努力实现“六个新跨越”。

　　（一）更加注重创新驱动，努力实现动能转换新跨越。坚持把创新摆在贵阳发展全局的核心位置，实施大数据战略行动，推进创新驱动发展战略，着力构建创新生态链，全面推进理念文化、体制模式、路径空间、技术产业、城镇乡村、社会事业和公共服务等创新，让创新无处不在、蔚然成风、开花结果。 通过五年努力，力争实现大数据的理论突破、立法突破、标准突破、治理突破、产业突破、安全突破，大数据技术和应用探索深入推进、政策法规先行先试、产业发展领先领跑，大数据成为经济发展加快转型、公共服务水平全面提高、社会治理能力快速提升的强大动力，建成大数据综合创新试验区。大众创业、万众创新氛围更加浓厚，众创、众包、众扶、众筹等支撑平台更加完善，知识、人才、资金、信息等要素高度集聚，形成一批有竞争力的创新型领军企业，新增国家级重点实验室1至2个，国家级工程技术研究中心2个，全社会研发支出占生产总值比重达到2.2%，高新技术制造业占工业增加值比重达45%以上，科技进步贡献率提高到65%以上，人才总量达到82万人。云岩区、南明区成为创新型中心城市的核心区，观山湖区成为创新型中心城市的主要功能发展区，花溪区、乌当区、白云区成为创新型中心城市的腹地，清镇市、修文县、息烽县、开阳县成为创新型中心城市的配套功能服务区和重要生态支撑。

　　（二）更加注重结构调整，努力实现产业转型新跨越。坚持走新型工业化道路，以大数据为引领，以“互联网+”行动计划、“中国制造2025”为路径，以打造大数据和大健康、山地旅游和都市现代农业、现代制造业和现代服务业“三个姊妹篇”为载体，推动产业融合、渗透发展，培育催生新技术、新产业、新业态、新模式，构建优势突出、特色鲜明，具有市场竞争力、可持续的现代产业体系。通过五年努力，大数据全产业链不断完善，大数据及其关联产业规模总量达到5000亿元；大健康产业形成千亿级产业集群；以山地为特色的世界旅游名城建设取得重大进展，形成南北呼应的2个国家5A级旅游景区精品集群，力争创建1个国家公园、1至2个国家级旅游度假区；现代农业增加值占一产增加值比重提高到45%，农产品加工产值突破1000亿元；现代制造业形成千亿级装备制造产业集群，成为全国重要的装备制造基地；现代金融、现代物流、商务会展等现代服务业加快发展，建成国内重要的呼叫中心与服务外包产业基地、区域性电子商务中心城市，力争建成西部科技金融创新城市、互联网金融创新城市和全国服务外包示范城市。

　　（三）更加注重先行先试，努力实现生态文明建设新跨越。坚定不移推进绿色发展，实施山水林田湖生态保护和修复工程，强化生态保护和环境治理，严格环境执法监管，守住“山上、天上、水里、地里”生态底线，确保建成空间开发格局更加优化、生态质量更加优良、城市环境更加宜居、绿色生产生活方式更加普及、生态文明制度体系更加完善的全国生态文明示范城市，探索形成可复制可推广的“贵阳路径”。到2020年，新增各类公园660个，全市公园达1000个以上，实现中心城区300米见绿、500米见园，环境空气质量稳步提升，集中式饮用水源地水质稳定达标，南明河水环境综合整治全面完成，国家水生态文明试点城市建设取得明显成效。新建一批循环经济示范基地和低碳工业园区，培育一批生态文明示范单位，全市产业低碳化、交通清洁化、建筑绿色化、主要污染物减量化、可再生能源利用规模化水平大幅提升。

　　（四）更加注重开放带动，努力实现对外合作新跨越。实施更加主动的开放战略，充分发挥四大平台在全省“1+7”开放创新格局中的引领和支撑作用，扩大生态文明贵阳国际论坛、数博会两张“名片”影响力，全面融入国家“一带一路”和长江经济带战略，深化与发达地区的区域合作，提高对外开放国际化水平，“全方位、宽领域、多层次”立体开放格局更加完善，内陆开放型经济新高地优势更加凸显，“十三五”末全市进出口总额突破200亿美元，实际利用外资超过23亿美元。坚持以开放倒逼改革、以问题倒逼改革，把供给侧结构性改革作为突破口，深入推进行政审批制度、国有企业、商事制度、资源配置体制机制和财税金融体制、投融资体制改革，科学配置劳动力、资本、土地、技术、管理等要素，显著提高供给体系质量和效益，激发市场主体活力和创造力，确保每年新增企业市场主体1000户以上，到2020年，非公有制经济占生产总值比重超过60%。

　　（五）更加注重统筹协调，努力实现城乡发展新跨越。按照中央城市工作会议的要求，更加科学地认识、尊重、顺应城市发展规律，统筹空间、规模、产业三大结构，统筹规划、建设、管理三大环节，统筹改革、科技、文化三大动力，统筹生产、生活、生态三大布局，统筹政府、社会、市民三大主体，框定总量、限定容量、盘活存量、做优增量、提高质量，形成符合贵阳实际，体现资源禀赋、文化特色的城市发展空间结构、规模结构、产业结构，努力建设山地特色现代化城市。通过五年努力，“双核多组团”城市发展格局进一步优化，“疏老城、建新城”取得重大进展，力争基本完成现有城镇棚户区改造，城镇空间布局和功能等级进一步提升，现代立体交通体系基本建成，工程性缺水问题基本解决，成为全国信息交换枢纽和信息存储中心，城市管理实现精细化、长效化、标准化，良好的市容环境卫生秩序成为常态，促进人口和要素自由流动的体制机制进一步形成，常住人口城镇化率达76%。城乡协调发展体制机制进一步健全，县域经济实力不断增强、小镇经济加快成为新的经济形态，城乡之间、区县之间居民收入水平、基础设施配套水平、基本公共服务均等化水平、人民生活水平等方面差距进一步缩小。大规模开展以“四在农家·美丽乡村”为主题的新农村建设行动，实施“六个小康”行动计划和农村环境综合整治，建成300个“美丽乡村”提高型示范点和20条以上示范带，推动城市、乡村、田园、社区各美其美、美美与共。

　　（六）更加注重普惠共享，努力实现小康建设新跨越。坚持发展成果共享，持续加大民生投入、夯实基层基础，努力增加优质公共产品和公共服务供给，让市民拥有更多的获得感、幸福感，建成更高水平的全面小康社会。通过五年努力，圆满完成大扶贫战略行动，确保每一个低收入家庭危房都能得到修缮，每一个低收入家庭都能吃得饱、穿得暖，每一个低收入家庭劳动力都有活干、有收入，每一个低收入家庭孩子都有学上、上得起学，每一个低收入家庭病人都能得到医疗救助，每一个农村低收入家庭的留守儿童、留守老人、留守妇女得到足够关爱。破解“民生十困”深入推进，教育发展、医疗事业、物价调控、社会保障、市容环境秩序、市民安全感等得到进一步提升。城市治理现代化水平不断提高，共治能力、善治能力、基层自治能力、法治能力、综治能力、德治能力等得到增强。文化特色进一步彰显，让我们这座城市更有文化厚度、更有人文温度！

　　三、2016年工作安排

　　今年是全面实施“十三五”规划的开局之年，是奋力打造创新型中心城市的起步之年，做好全年各项工作意义十分重大。

　　今年全市经济社会发展的主要预期目标是：生产总值增长11%，规模以上工业增加值增长10%左右，固定资产投资增长18%，社会消费品零售总额增长11%，外贸进出口总额增长11%，公共财政预算收入增长10%左右，城镇、农村常住居民人均可支配收入分别增长10%左右和11%左右，居民消费价格涨幅、节能减排指标控制在省下达目标范围内，空气质量稳定在全国省会城市前列。

　　围绕上述目标，我们将认真贯彻落实中央经济工作会议和全省经济工作会议精神，按照市委的部署，在十个方面精准发力，确保“十三五”经济社会发展开新局、迈新步。

　　（一）在转方式上开新局、迈新步。紧紧围绕打造创新型中心城市，加大创新力度、提高创新速度、拓展创新维度，加快形成以创新引领发展、驱动发展的良好格局。一是建强创新平台，大力推进国家级大数据产业发展集聚区和大数据产业技术创新试验区建设，申报筹建公共大数据国家重点实验室，与全国信息技术标准化技术委员会大数据工作组合作开展3项贵阳标准研制、2项国家标准研制与试点示范。抓好“一站一台”“一部一院”和高校科研院所科技创新联盟等科技平台全方位推广应用，引导企业加大研发投入，推进政产学研用合作升级，与北京科技成果及专利技术合作达30项以上，实施重大科技创新成果转化项目10个，新增国家级研发机构2个、省级研发机构5个，全社会研发投入达35亿元。二是激发创新活力，实施众创空间建设培育工程和“筑巢引凤”计划、黔归人才计划、大数据人才培养引进工程，支持“新三线”咖啡、联合智造、启林创客小镇等一批示范性和创新型孵化器做大做强，加快建设“中国西部众创园”，引进各类高层次创新创业人才600名以上，聚集国内外创客团队100个以上，带动虚拟空间聚集创客5万人。三是优化创新资源配置，抓紧国家自主创新示范区试点政策的推广落地，用好研发费用加计扣除等税收政策，以政府“小投入”撬动全社会“大投入”。继续实施“创客十条”，完善“3个20万”配套政策，着力构建容错试错机制，支持“双创”“四众”，使贵阳成为创客“筑梦”、贵漂“圆梦”的乐园。

　　（二）在推改革上开新局、迈新步。按照“十个更加注重”的要求，围绕投资有回报、产品有市场、企业有利润、员工有收入、政府有税收、环境有改善，扎实抓好供给侧结构性改革。一是去产能，加大政策引导力度，完善企业退出机制，实施“双退出”行动计划。更加注重运用市场机制、经济手段、法治办法化解产能过剩，对那些已停产半停产、连年亏损、资不抵债、靠政府补贴和银行续贷存在的企业，进行兼并重组或依法破产。二是去库存，进一步优化城市开发模式，完善土地供给方式，发展住房租赁市场，结合解决拆迁安置房、呼叫中心配套用房等，统筹推进房地产去库存化。大力发展养老地产、医疗地产、旅游地产等新兴业态，促进房地产业平稳健康发展。三是降成本，以大数据推动简政放权、放管结合、优化服务改革，加快行政审批标准化建设和各级公共服务平台建设，开展降低实体经济成本行动，深入实施“百千万”工程和民营经济服务年行动，用好市中小企业云服务平台，建立小微企业信贷风险补偿金，设立民营企业转贷应急周转资金，帮助企业降低制度性交易、税费、人工、物流、财务等成本。四是强企业，认真落实全省“百企引进”“千企改造”“双培育”工程，年内引进世界500强企业或跨国公司5家，实施千万元以上重大技术改造项目30个，新增规模以上工业企业、限额以上商业企业各100户，规模以上服务业企业100户以上。新注册商标超过4000件，新增著名商标、知名商标均达30件，力争培育中国驰名商标2至3件。同时，统筹推进国有企业、社会建设、生态文明建设等领域的改革。

　　（三）在稳增长上开新局、迈新步。投资仍然是拉动经济增长的第一动力，要更好地发挥投资对增长的关键作用和消费对增长的基础作用，激发新活力，释放新需求，创造新供给，推动经济平稳健康发展。一是保持有效投资力度。编制完善“十三五”投资项目库，项目投资规模达2万亿元以上，争取一批项目列入国家西部大开发“十三五”规划。积极推广PPP模式，加大股权融资、债券融资力度，扩大直接融资比重，不断提高融资能力，优化投资结构。全市245个重大工程重点项目完成投资1200亿元，固定资产投资完成3250亿元，产业投资占比超过36%，民间投资占比超过50%。二是持续壮大消费需求。实施促进消费三年行动计划，积极促进旅游消费、壮大养老健康消费、稳住住房和汽车等大宗消费、扩大信息消费、增加中高端消费。推进便利消费进社区、便利服务进家庭“双进”工程，大力打造15分钟社区便民服务圈。优化提升大南门、大西门、喷水池等传统商圈，加快培育西南商贸服务业集聚区、中天未来方舟、花果园等新型商圈，建成新市西滨河商业街区、汇金购物中心，社会消费品零售总额达到1177亿元。三是努力扩大对外贸易。充分发挥贵阳综保区的功能作用，培育壮大跨境电商、展示交易、保税物流、出口加工、服务外包等业态，积极开拓东盟、南亚和南美等新兴市场,力争全市进出口总额突破120亿美元。

　　（四）在调结构上开新局、迈新步。把打造“三个姊妹篇”作为我市产业结构调整的主战场，不断提高经济发展质量和效益，推动产业发展迈向中高端水平。一是打造大数据和大健康“姊妹篇”。1.全力实施大数据战略行动，围绕打造大数据全产业链，实施全域公共免费WiFi城市二期工程，建成政府数据开放和共享交换平台，加快大数据加工清洗产业基地、大数据安全产业基地、大数据软件园和交通大数据孵化器建设，推进惠普、世欣蓝汛等数据中心建设，抓好窦官、红华呼叫产业园、科大讯飞等呼叫和服务外包项目，力促富士康液晶面板项目开工，支持朗玛、货车帮等龙头企业做大做强，确保京东电商产业园全面投用，全市呼叫中心坐席规模达到15万席，服务外包合同签约金额突破8亿美元，电子商务交易额突破600亿元，大数据及其关联产业规模总量达到1300亿元、增长35%。2.全力实施千亿级大健康产业集群发展计划，围绕“医、养、健、管”，以筹办省第二届大健康医药产业发展大会为契机，培育产业集群、打造重点平台，全年新开工大健康产业项目48个，累计完工64个。支持乌当建设大健康医药产业引领示范区，加快益佰工业园等园区建设，确保同济堂骨科中成药生产基地、汉方集团中成药现代化生产基地、智汇云锦大健康医药产业示范基地等建成投产，力争泰邦生物、拜特制药发展成为十亿级企业。加快推进大数据在大健康领域的运用，支持贵阳互联网医院发展和益佰制药、同济堂药业等企业开展大数据研发应用。二是打造山地旅游和都市现代农业“姊妹篇”。1.山地旅游方面，围绕建设世界旅游名城，打好生态牌，打造精品化核心吸引物，力争国家5A级旅游景区创建工作取得重大突破，新增2至3个国家4A级旅游景区，加快花溪文化旅游创新区建设，确保天河潭景区在十一黄金周全新亮相。策划推出一批世界级观光线路，引进国际知名度假品牌酒店3家以上，新评定避暑度假精品酒店5家。完成IBM——太极贵阳智慧旅游大数据联合创新中心平台搭建。全市旅游接待人次、旅游总收入分别增长18%和20%。2.都市现代农业方面，推进对台农业全面合作，建成一批合作示范园，新增省级农业园区4个，新增新型农业经营主体130个。大力发展农村电商，实现行政村电商便民服务店全覆盖，打通“黔货出山”和“网货下乡”的双向通道。促进一二三产融合发展，打造一批农旅综合体、美丽乡村旅游度假区、观光农业园区和休闲农业示范点，办好农业嘉年华活动。支持修文猕猴桃产业、开阳茶产业加快发展。三是打造现代制造业和现代服务业“姊妹篇”。1.大力发展现代制造业，强力推进吉利新能源汽车项目，开工华旭汽车电子产品生产线、贵州雅光汽车电子元器件生产线等项目。在装备制造、智能制造、磷煤化工、铝及铝加工等产业中，推广应用一批新技术、新装备、新流程，启动建设中铝贵州分公司煤电铝一体化项目，确保开磷集团多功能磷复肥及精细磷化工装置技术改造项目投产。对老干妈、黔五福等重点企业加大扶持力度，着力培育百亿级、十亿级现代食品加工企业。全市重点特色工业产业实现增加值620亿元左右。加快推进制造业由生产型向生产服务型转变，支持有条件的制造业企业面向市场提供服务，促进技术装备、技术服务价值提升。着力推进军民融合发展战略，促进军民协同创新。2.大力发展现代服务业，以大数据金融产业园为载体，推动新兴金融业态健康发展，深入开展全国移动金融试点工作，大力发展大数据金融、众筹金融，贵阳大数据金融交易所、众筹金融交易所年交易额分别突破5亿元和10亿元。加快完善贵州金融城配套设施，入驻金融机构达25家。贵阳银行实现全省县级分支机构全覆盖。坚持生产性服务业和生活性服务业两手抓，建成全市物流大数据中心，办好2016阿里巴巴“年货节”，组织好购物节、车展、房展以及文化旅游等促销活动。推进现代服务业集聚区建设，确保西南商贸服务业集聚区、贵阳火车北站商务区、贵阳大数据服务产业基地等亮出新形象。

　　（五）在优生态上开新局、迈新步。大力实施“公园城市”工程，纵深推进“蓝天”“碧水”“绿地”“清洁”“田园”五项保护计划，开展生态修复和城市修补“双修”工作，提升生态环境，推动绿色发展。一是以更高标准抓建设。新建各类公园249个，重点打造27个示范公园，新增公园面积2000公顷以上。实施中心城区108个山体生态修复工程，修复植被960余亩。深入推进“绿美贵阳”行动计划，完成营造林18万亩以上，全市森林覆盖率达46%以上。大力开展通道绿化，启动中心城区屋顶绿化，提升环城林带森林质量，保护好城市的“绿色项链”。二是以更大力度抓治理。深入开展大气污染防治，稳定提高环境空气质量，力争在全国74个重点城市排位前移。大力推进“两湖一库”保护工程，加快建设贵州清镇红枫湖国家湿地公园，开展阿哈水库流域综合治理，加快南明河水环境综合整治二期工程，建设孟关等6座污水处理厂，完成三江口等3个湿地工程。推进城乡黑臭水体整治。开展土壤污染防治行动。三是以更严制度抓监管。建立自然资源资产不动产登记机构和登记管理制度。实施生态环境保护网格化监管，完善环境信用评价制度，大力推行环境污染强制责任保险，积极推广第三方治理。建立用能权、用水权、排污权、碳排放权初始分配制度。建设市节能低碳在线监测平台，完善节能降耗统计、监测和考核指标体系。建立生态保护约谈制度，用好环境保护“六个一律”和森林保护“六个严禁”两把“利剑”，以零容忍的态度严厉打击环境违法行为。

　　（六）在扩开放上开新局、迈新步。坚持高端化、国际化，努力以大开放促进大发展，加快打造内陆开放型经济新高地。一是狠抓开放平台升级，着力提升“1+4”开放实体平台和“2+N”开放活动平台，加快中关村贵阳科技园和高新区、经开区、贵阳综保区、航空港经济区建设，办好生态文明贵阳国际论坛2016年年会、数博会和众筹大会、中国——东盟教育周、酒博会等重大活动。高新区围绕“一体四区”战略定位，加快打造千亿级开发区；经开区以智能制造为主攻方向，加快打造全省首批千亿级园区；综保区围绕面向国际开放桥头堡的目标，着力发展跨境电商、离岸服务外包、离岸数据服务、离岸结算、融资租赁、加工贸易、配套物流等产业，让业务量大幅提升；航空港经济区骨干路网年内基本建成，园区配套设施建设有较大进展，加快形成航空指向性明显的航空港产业链和现代产业体系。二是狠抓对外合作升级，在抓好京筑创新驱动区域合作等基础上，以大数据为重点，以融入“一带一路”国家战略为契机，深化与发达国家和周边国家的合作，力争启动中韩合作产业园、中印合作产业园建设，加快《中瑞自由贸易协定》贵阳示范园等建设，支持企业向外输出资本、技术、装备和服务，对外承包工程完成营业额2亿美元、增长25%。三是狠抓招商引资升级，精心筹办2016云上贵州·大数据招商引智再出发等活动，以欧美发达国家为重点，组织开展系列国际招商推介、招才引智活动，新引进十亿级项目10个，力争在引进百亿级项目上取得突破，全年实际直接利用外资超过11亿美元，省外到位资金超过3100亿元。

　　（七）在美城乡上开新局、迈新步。把创造优良人居环境作为城镇化工作的中心目标，围绕构建城乡发展新形态，以“疏老城、建新城”和“四在农家·美丽乡村”建设为重点，统筹城乡规划建设管理，促进城乡协调发展。一是在优化空间布局上统筹。推进“多规合一”，开展《贵阳市城市总体规划（2011——2020年）》修改和《贵阳市土地利用规划（2006——2020年）》调整完善工作，划定城市开发边界，加强控制性详规编制，提高城市设计水平。加大棚户区改造力度，提高货币化安置比例，全年改造城镇棚户区3万户，推进贵州大学、贵州商学院、贵州财经大学等高校老城校区向外搬迁，加快建设三马、孟关、东风等城市组团，配套实施图书馆、音乐馆、科技馆等功能性设施项目。高标准编制村镇规划，合理布局村寨、园区、公共服务设施，力争实现全覆盖。二是在产业发展上统筹。以“八个一批”项目为抓手，推动县域经济加快发展，规划建设一批数字小镇、创客小镇、众筹小镇、绿色小镇、风情小镇和乡愁小镇，大力发展小镇经济。推进以高新区、综保区为龙头的北部高新技术产业带和以经开区、航空港经济区为龙头的南部现代制造业产业带建设，加快区县优势互补、融合发展。建成北部产业带联接大道。三是在基础设施建设上统筹。建成1.5环，同步推进BRT（快速公交）系统，加快轨道交通1号线和2号线一期建设，启动2号线二期和S2号线一期北段控制性土建接驳工程，开展3号线前期工作。加快推进西南商贸服务业集聚区综合交通枢纽项目，抓好贵阳与贵安“六纵六横”区域一体化路网建设。积极推进贵阳至遵义复线、息烽至黔西、开阳至息烽等高速公路项目，贵阳至南宁、贵阳至兴义等铁路开工建设，沪昆高铁贵阳至昆明段建成通车。推进海绵城市建设，实施宾阳大道等城市综合管廊工程。大力建设光网城市，实现城市光纤入户率超过90%、乡村100M光纤宽带覆盖率达80%，出省带宽达4000G，力争建成国家级互联网骨干直联点。以“六个小康”建设为重点，实施城乡一体化发展三年攻坚计划，推进城镇基础设施向农村延伸，提等改造农村道路500公里，开工建设红岩水库主体工程，确保那卡河水库大坝主体工程完工。四是在管理治理上统筹。围绕“衣食住行、生老病死、安居乐业”12个字加强城市管理和服务，改革城市管理体制，运用大数据手段提升城市管理水平，着力整治交通堵点、垃圾臭点、排涝塞点、经营乱点、信号盲点，强化水、电、气、讯、运等要素保障，提升防洪、排涝、山体滑坡、公共安全等治理水平。把文化元素充分融入城乡规划建设管理，“里子”“面子”一起要，“颜值”“气质”一起抓，打造一批特色街区，加快改造提升中华路等城市干道，扩大“示范街、严管街”范围，集中整治提升181条背街小巷道路，实施17个增绿添彩工程及立体绿化工程，完成49个农贸市场升级改造和300个老旧小区院落改造提升。推进高雁、比例坝垃圾填埋场技改升级，扩建、改造一批小型垃圾中转站。深入开展农村环境综合整治，完成58个乡镇、223个村环境综合整治任务，新增5条美丽乡村示范带，创建40个“提高型”示范点。市委、市政府决定，从今年起，连续三年每年筹资100亿元以上用于“三农”工作。五是在配套改革上统筹。把促进有能力在城镇稳定就业和生活的常住人口有序实现市民化作为首要任务，加强农业转移人口市民化的战略研究，统筹推进户籍制度改革和土地、财政、教育、就业、医疗、养老、住房保障等领域配套改革。深化农村产权制度改革，维护好进城落户农民的土地承包经营权、宅基地使用权、集体收益分配权，调动农业转移人口进城落户的积极性。

　　（八）在铸文化上开新局、迈新步。大力实施“文化惠民”工程，弘扬“天人合一、知行合一”人文精神，加快建设“三足鼎筑”精神大厦。一是突出打造阳明文化品牌。珍视阳明文化资源，深度挖掘阳明心学时代价值。大力推进以阳明洞为核心的阳明文化园建设，确保年内亮相。加快推进以阳明祠为核心的历史文化街区规划建设工作，筹备建设阳明文化公园。大力发展孔学堂事业，加快三期项目建设，完成阳明先生纪念馆改扩建工程。办好阳明文化节，推出一批以阳明文化为主题的文艺精品。二是深入打造生态文化名片。继续开展资源环境市情教育和生态价值观教育，推进生态文明建设细胞工程，新创建一批生态文明机关、单位、企业、医院、学校、社区、家庭。丰富生态文化内涵和外延，举办“春雨工程”等公共文化活动和潮流音乐周等时尚文化活动。完成第二批国家公共文化服务体系示范区创建任务。三是积极打造民族文化亮点。加强历史文化名镇、名村和民族村寨、传统村落保护，推进非物质文化遗产的保护、发展、传承和合理利用，培育一批民族民间文化名品、名师、名企。四是大力发展文化产业。推进省“十大文化产业园”“十大文化产业基地”贵阳项目建设，抓好时光贵州、乡愁贵州、多彩贵州等文化综合体项目，大力打造“贵山秀水忆乡愁”“黔茶飘香·筑城品茗”等文化品牌。力争文化产业增加值达110亿元。

　　（九）在惠民生上开新局、迈新步。持续抓好民生解困，办好民生实事，不断提高群众幸福指数。大力实施大扶贫战略行动,率先建成大数据精准扶贫平台，率先在全省实现低保线和脱贫线“两线合一”，农村低收入困难群体年人均可支配收入整体越过4300元。抓好省内结对帮扶工作，帮助9个重点贫困县打好脱贫攻坚战。纵深推进“教育立市”战略，全面改造薄弱学校10所，新建改扩建公办幼儿园10所、中小学8所，新增学位5000个，加快农村寄宿制学校标准化建设，大力发展普惠性学前教育，加快义务教育优质均衡发展，提高高中阶段教育质量，创建3所优质中职学校，继续推进贵州（清镇）职教城建设，促进高等教育内涵发展和优质民办教育发展，在全省率先实现县域义务教育基本均衡和基本普及十五年教育。着力建设健康城市，全面深化公立医院综合改革，启动建设贵阳中医院、市公共卫生救治中心，建成市一医分院、口腔医院新楼等项目，做强市医院管理（集团）有限公司，标准化建设社区卫生服务中心6个、乡镇卫生院7所、村卫生室60个，落实一对夫妇可生育两个孩子政策。抓好南明河沿线等全民健身步道建设，举办2016贵阳国际马拉松赛、贵阳国际拳击季等品牌赛事，打造夏季马拉松名城。抓好“保供稳价”，高标准新建蔬菜保供基地1万亩，建设惠民生鲜超市6个和社会化大型生鲜超市6个。落实就业优先计划，推进全民创业行动，新增城镇就业22.5万人，农村劳动力转移就业3.25万人，城镇登记失业率控制在4.2%以内。实施社会保障兜底工程，推进城乡社会保障全覆盖，更加关心低收入群体和困难群众的生产生活，健全政府、社会、家庭“三位一体”的关爱服务救助保护体系。加大公租房配套基础设施建设力度，确保按时投入使用。推进农村敬老院、农村幸福院和县级养老示范项目建设。

　　（十）在促和谐上开新局、迈新步。进一步创新基层管理体制，强化社区主体责任，做强居（村）委会，做精网格，形成基层社会治理多元化格局。健全城市公共安全体系和应急管理体系，高标准做好地质灾害隐患排查治理，深入开展城市老旧住房排查整治，完善高楼消防安全设施和防灾措施，持续开展交通、煤矿、消防、危险化学品等重点行业领域安全生产专项整治行动，加大食品药品安全监管力度，深入开展“两严一降”、禁毒人民战争，强化社会治安立体防控体系建设，抓好信访维稳“一号工程”，严格落实重大决策社会稳定风险评估制度，推进公共法律服务体系建设，严厉打击非法集资，全力防范经济风险、社会风险、自然风险，促进社会和谐稳定，给市民群众一个稳稳的安全感。

　　继续推进国防、人防、国家安全、审计、司法行政、民族宗教、移民、地方志、档案、侨务、对台、仲裁、气象、老龄、慈善、残疾人、爱国卫生等事业发展，支持法院、检察院工作，支持工青妇、关工委、红十字会、计生协会等组织做好工作。

　　四、扎实抓好政府自身建设

　　认真践行“三严三实”，以忠诚为魂、人民为本、法治为先、担当为要、廉洁为基，努力建设组织放心信任、人民满意信赖的政府。

　　（一）以忠诚为魂。始终把对党绝对忠诚作为政府干事创业、做好工作的思想灵魂。强化看齐意识，坚持理想信念的“高线”，严守党的政治纪律和政治规矩，切实做到心中有党、心中有民、心中有责、心中有戒，坚决维护中央权威，坚定不移贯彻党的路线、方针、政策，坚决服从省委、省政府和市委领导，不折不扣执行各项决策部署，切实将市委的一切主张、一切意图转化为具体工作、具体举措，以有力有效的结果证明市委决策的正确性。

　　（二）以人民为本。始终把增进人民福祉、促进人的全面发展作为政府工作的出发点和落脚点，不断强化宗旨意识和以人民为中心的发展思想，按照人人参与、人人尽力、人人享有的要求，充分调动人民群众的积极性、主动性和创造性，问计于民，问需于民，顺应群众期盼，回应群众关切，把一切工作做到百姓心坎上。全面接受人民监督，充分体现人民意愿。

　　（三）以法治为先。始终坚持把依法行政作为政府工作的生命线，自觉运用法治思维和法治方式，加快法治政府建设。牢记政府是国家权力机关的执行机关，严格履行宪法和法律赋予的职能职责，自觉接受人大法律监督、政协民主监督，以及群众监督、舆论监督，高度重视民主党派、工商联和无党派人士的意见建议，充分发挥工青妇等人民团体的桥梁纽带作用，同心同德做好工作。

　　（四）以担当为要。始终把敢于担当作为政府自觉，铸就一副铁肩，切实担起使命、担起责任、担起风险，攻坚克难、动真碰硬，以大担当担起大事业、推动大发展。坚持一分部署、九分落实，充分发扬“钉钉子”的精神，敢作敢为、善作善成，持续努力、久久为功，靠本事吃饭，拿数据说话，创造出经得起历史和人民检验的工作实绩。

　　（五）以廉洁为基。始终把廉洁从政作为政府工作的底线，坚定不移把反腐倡廉推向深入。严格落实党风廉政建设主体责任，从严从实执行《中国共产党廉洁自律准则》《中国共产党纪律处分条例》，严格遵守中央、省委和市委各项规定，坚持不懈纠正“四风”，运用大数据手段监督权力，实现“数据铁笼”全覆盖，织密扎紧“制度笼子”，切实管好公务人员、公共资源、公共权力、公共资金，做到“人在干、云在算、天在看”，以实际行动树立为民务实清廉的良好形象。

　　各位代表！蓝图已经绘就，号角已经吹响！让我们紧密团结在以习近平同志为总书记的党中央周围，在省委、省政府和市委的坚强领导下，坚定不移守底线、脚踏实地走新路，全面打造贵阳发展升级版，做高铁路上的“火车头”、战斗机上的“发动机”，为打造创新型中心城市，建成大数据综合创新试验区、全国生态文明示范城市、更高水平全面小康社会而努力奋斗！
